# Multiomics profiling of transcriptional and metabolic responses of *Bacillus siamensis* LSZ23 to corn straw

**DOI:** 10.3389/fmicb.2026.1935834

**Published:** 2026-09-22

**Authors:** Wei Zhang, Gongfu Shi, Wenqing Cao, Xi nhui Huang, Tianjiao Zhang, Huimin Shi, Shuli Wei, Xinlan Wang, Yujie Lin, Zhanyuan Lu, Shaofeng Su, Xiaoqing Zhao

**Affiliations:** 1 School of Life Sciences, Inner Mongolia University, Hohhot, China; 2 Key Laboratory of Black Soil Protection and Utilisation (Hohhot), Inner Mongolia Academy of Agricultural and Animal Husbandry Sciences, Ministry of Agriculture and Rural Affairs, Hohhot, China; 3 Key Laboratory of Degradation Farmland Ecological Restoration and Pollution Control, Soil Microbial Germplasm Bank in the Pastoral Interlaced Region of Northern China, Hohhot, China

**Keywords:** *Bacillus siamensis*, carbohydrate-active enzymes, cellulase, lignocellulose degradation, multiomics

## Abstract

Agricultural straw is an abundant yet underutilised lignocellulosic resource whose microbial valorisation requires cellulolytic strains that sustain enzyme production under substrate-imposed stress. Although *Bacillus siamensis* is valued for plant growth promotion and biocontrol, its lignocellulose-degrading capacity and regulation remain largely unexplored. *B. siamensis* LSZ23, isolated from humic soil in Inner Mongolia, China, produced a balanced cellulolytic system (CMCase 0.321, β-glucosidase 0.275, avicelase 0.240 and FPase 0.209 U/mL in 24 h LB culture) and reduced sterilised corn straw dry mass by 19.5% within 20 d in solid-state culture (10^9^ CFU/g), with scanning electron microscopy revealing extensive fibre-bundle disruption. Its 4.12 Mb genome (46% GC, 4,120 protein-coding genes) encodes 570 predicted carbohydrate-active enzyme (CAZyme) genes, with marked expansion of glycoside hydrolase family GH1, and harbours 31 orthogroups absent from four reference genomes, notably oligosaccharide ATP-binding cassette transporters and starch-processing enzymes. Upon straw exposure, transcriptomics (173 differentially expressed genes) and metabolomics (1,094 differentially abundant metabolites) indicated coordinated reprogramming rather than simple enzyme amplification: motility and chemotaxis genes were repressed, the biofilm-matrix gene *tapA* was induced, carbon uptake shifted transcriptionally towards a phosphotransferase system-mediated cellobiose route, and the glutamine synthetase-glutamate synthase pathway and *de novo* purine biosynthesis were downregulated, consistent with reduced high-cost anabolism. Purine metabolism was jointly significant across both omics. Lignin-derived phenolics accumulated alongside five upregulated MarR-family transcriptional regulators, although their ligand-regulon relationships remain undetermined. These findings provide a multiomics framework for LSZ23-mediated lignocellulose bioconversion and identify it as a candidate hydrolytic partner for straw degrading consortia.

## 1 Introduction

Lignocellulosic crop residues constitute one of the largest renewable biomass streams on the planet and China ranks among their foremost producers. According to the Ministry of Agriculture and Rural Affairs, national straw output reached approximately 870 million tonnes in 2024, of which about 730 million tonnes were collectable, while the comprehensive utilisation rate was sustained above 88% (Ministry of Agriculture and Rural Affairs of the People’s Republic of China [MARA], 2025). Structurally, straw is built mainly from three polymers, cellulose, hemicellulose and lignin, whose proportions vary with crop type but typically fall within 38%–50%, 23%–32%, and 15%–25%, respectively. Linked by covalent and non-covalent bonds, these components assemble into a compact three-dimensional network that strongly resists enzymatic hydrolysis. Conventional handling routes, notably open-field burning and burial, release considerable greenhouse gases and can introduce phytopathogens into farmland soils, running counter to the goals of green, low-carbon agriculture (Han et al., 2024; Mao et al., 2025). Among the available alternatives, biological degradation mediated by microorganisms has emerged as the most promising approach owing to its high substrate specificity, cost-effectiveness and environmental compatibility (Wang et al., 2021).

The enzymatic deconstruction of cellulose, the primary structural polysaccharide of straw cell walls, is mediated by a cooperative enzyme system requiring at least three complementary activities acting in concert. Endoglucanases (EC 3.2.1.4) cleave β-1,4 linkages at random within the disordered amorphous regions, creating additional free ends; cellobiohydrolases (exoglucanases; EC 3.2.1.91) then act processively on the reducing and non-reducing ends of crystalline cellulose, successively liberating cellobiose; finally, β-glucosidases (EC 3.2.1.21) convert cellobiose to glucose and thereby remove the end-product that would otherwise exert feedback inhibition on the two upstream activities (Lynd et al., 2002; Datta, 2024). Because these enzymes belong to the carbohydrate-active enzyme (CAZyme) superfamily, the extent to which their secretion is coordinated constitutes a principal bottleneck governing the rate of straw breakdown, thereby shaping the transfer of carbon and nitrogen into soil biogeochemical cycling (Drula et al., 2022; Hsin et al., 2025). Although filamentous fungi, such as Trichoderma reesei, have historically served as model reference strains for industrial cellulase production, their performance is constrained by relatively narrow environmental tolerance ranges, slow growth kinetics, significant end-product inhibition and reduced performance under high substrate loadings typically encountered in industrial settings (Kant et al., 2024). These limitations have intensified the search for cellulolytic bacteria as alternative catalysts, given their rapid growth rates, broad tolerance to pH and temperature, amenability to genetic manipulation and capacity to maintain stable enzyme production under variable environmental conditions (Mao et al., 2025).

Within the genus *Bacillus*, members of the *Bacillus subtilis* species complex, including *Bacillus velezensis*, *Bacillus amyloliquefaciens* and *Bacillus siamensis*, have attracted particular attention as candidates for agricultural biotechnology. These aerobic, endospore-forming, Gram-positive bacteria are ubiquitous in soil and plant environments and produce a broad spectrum of secondary metabolites, hydrolytic enzymes and biosurfactants (Chun et al., 2019). *B. velezensis*, among the most studied members of this complex, has been documented to harbour extensive CAZyme repertoires and exhibit notable cellulolytic performance, as exemplified by previously characterised strains whose degradation mechanisms have been explored through genomic analyses (Zhang et al., 2026). In contrast, *B. siamensis*, which shares > 99% 16S rRNA gene sequence identity with *B. velezensis*, has been reported to promote plant growth through phosphate solubilisation, biocontrol of phytopathogenic fungi and induction of systemic resistance (Dong et al., 2023; Sun et al., 2024). Pan-genomic analyses of the *B. amyloliquefaciens* group further revealed that *B. siamensis* harbours distinctive genomic features, including unique biosynthetic gene clusters and accessory metabolic functions that are not shared with other members of the complex (Chun et al., 2019). Although this accumulating evidence underscores the agronomic versatility of *B. siamensis*, how *B. siamensis* degrades lignocellulose and which regulatory machinery drives that activity have received little attention to date.

A central challenge in utilising cellulolytic bacteria for agricultural straw valorisation is not only to identify strains with high enzymatic activities but also to understand how they dynamically coordinate enzyme biosynthesis, substrate transport, energy allocation and cellular stress responses within the chemically complex microenvironment of decomposing straw. Agricultural straw presents microbes with at least two additional and interconnected physiological challenges beyond lignocellulose recalcitrance. First, the inherently high carbon-to-nitrogen (C:N) ratio of plant biomass (typically greater than 70:1) imposes severe nitrogen limitation on microbial growth, forcing cells to compromise between biomass expansion and catabolic enzyme production (Sun et al., 2022; Zhang et al., 2025). Second, as lignocellulose depolymerisation proceeds, a chemically heterogeneous array of lignin-derived phenolic and aromatic compounds, including ferulic acid derivatives, hydroxycinnamic acids and phenylpropanoids, accumulates within the substrate environment, many of which are cytotoxic to bacteria (Sánchez, 2009; Li et al., 2024). Survival and efficient hydrolysis of lignocellulose under such dual stresses require microorganisms to maintain a precise physiological balance between energy-intensive enzyme secretion, targeted detoxification and maintenance of essential cellular functions.

Traditional approaches that rely solely on genomic analysis or enzyme activity assays capture only fragmentary, condition-specific information about a strain’s catabolic potential, providing limited insight into the dynamic regulatory mechanisms governing degradation performance under substrate-imposed stress. Multiomics frameworks, which integrate whole-genome sequencing with transcriptomics and metabolomics, enable a system-level dissection of how genetic potential is translated into metabolic flux and phenotypic output under defined substrate conditions (An et al., 2021; Xie et al., 2024). Transcriptomics reveals genes that are actively induced or repressed during cellulose exposure, thereby illuminating the hierarchical regulatory architecture governing CAZyme expression and carbon catabolite repression. Metabolomics documents the downstream biochemical consequences of transcriptional reprogramming, revealing the metabolic intermediates and flux distributions that determine degradation efficiency. The integration of these two data streams through joint Kyoto Encyclopedia of Genes and Genomes (KEGG) pathway enrichment analysis further characterises coordinated gene and metabolite regulatory modules that no single approach could reveal in isolation (Fang et al., 2025). Such integrated multiomics characterisation has been successfully applied to elucidate lignocellulose degradation by *Cellulomonas iranensis* CI1 (Chen et al., 2025) and lignin metabolism in *B. amyloliquefaciens* MN13 (Li et al., 2024), yet such systematic multiomics characterisation of *B. siamensis* remains entirely absent from the literature.

In this study, strain LSZ23, identified as *B. siamensis*, was recovered from humic soil in Inner Mongolia, China. To dissect the molecular basis of its bioconversion capacity under the physicochemically heterogeneous conditions imposed by lignocellulosic substrates, we adopted a multiomics framework coupling whole-genome sequencing with comparative genomics, transcriptomics and metabolomics. Specifically, this study aimed to (i) characterise the genomic architecture of LSZ23, with emphasis on its CAZyme repertoire and the gene content that distinguishes it from the selected reference genomes; (ii) delineate the global transcriptional and metabolic responses elicited by corn straw exposure, focusing on the coordination between carbohydrate transport, glycolytic pathways and energy partitioning under straw-amended conditions; and (iii) characterise the transcriptional and metabolic signatures associated with the tolerance and catabolism of lignin-derived aromatic compounds, including the concurrent induction of MarR-family regulators during active straw degradation. Collectively, these findings provide an integrated genetic and physiological framework for understanding LSZ23-mediated lignocellulose bioconversion and establish a theoretical foundation for its use as a microbial agent in agricultural straw valorisation.

## 2 Materials and methods

### 2.1 Isolation and screening of lignocellulose-degrading bacteria

Humic soil samples were collected from Arong Banner, Hulunbuir City, Inner Mongolia, China (48°8′39″ N, 123°28′19″ E). A 5 g portion of soil was resuspended in 45 mL of sterile water and agitated at 180 rpm for 30 min, after which a tenfold dilution series spanning 10^–1^ to 10^–9^ was prepared. Triplicate aliquots of the 10^–7^, 10^–8^, and 10^–9^ dilutions were spread onto sodium carboxymethylcellulose (CMC-Na) agar plates (containing 10.0 g/L CMC-Na, 1.0 g/L K_2_HPO_4_, 1.0 g/L NH_4_NO_3_, 0.02 g/L CaCl_2_, 0.2 g/L MgSO_4_⋅7H_2_O, and 0.05 g/L FeCl_3_⋅6H_2_O) and incubated at 30 °C for 24 h. Cellulolytic colonies were identified by staining the plates with 0.2% (w/v) Congo red solution for 15 min, followed by destaining with 1 mol/L NaCl for 15 min; colonies producing clear hydrolysis halos were selected for further characterisation (Teather and Wood, 1982).

Selected isolates were subsequently streaked onto three indicator media to determine their Hydrolysis Capacity Ratio (HCR): Avicel^®^ microcrystalline cellulose medium (Product No. PH101, Sigma Aldrich), CMC-Na medium and Aniline blue medium. HCR was expressed as the diameter of the hydrolysis halo divided by that of the corresponding colony (Prem Anand et al., 2010). Strains exhibiting the highest HCR values were selected for subsequent characterisation. Pure cultures were obtained by repeated streaking and microscopic verification and stored at −80 °C in Luria–Bertani (LB) broth (tryptone 10 g/L, yeast extract 5 g/L, NaCl 10 g/L) containing 30% (v/v) glycerol.

### 2.2 Determination of cellulase activity and strain characterisation

#### 2.2.1 Determination of cellulase activity

Strains were cultured in LB medium at 30 °C and 170 rpm for 24 h (inoculated at 3% v/v from an overnight preculture). The cell density of the 24 h LB culture was quantified by ten-fold serial dilution in sterile distilled water, spread-plating of 100 μL aliquots onto LB agar, incubation at 30 °C for 24 h and colony counting, which confirmed a density of approximately 1.0 × 10^8^ CFU/mL (OD_600_ 0.9–1.2, *n* = 5 independent cultures). Cells were removed by centrifugation (15,300 × *g*, 10 min, 4 °C) and the resulting cell-free supernatants were taken as crude enzyme preparations. The four cellulase components FPase, CMCase, avicelase, and β-glucosidase were assayed following Ghose (1987). For each assay, 0.5 mL of crude enzyme solution was mixed with the respective substrate in 0.05 mol/L sodium citrate buffer (pH 4.8) and incubated at 50 °C. FPase activity was determined using 50 mg Whatman No. 1 filter paper in 1.0 mL buffer for 60 min. CMCase activity was assayed using 0.5 mL of 2% (w/v) CMC-Na for 30 min. Avicelase activity was assayed using 0.5 mL of 1% (w/v) Avicel for 60 min. β-Glucosidase activity was assayed using 0.5 mL of 1% (w/v) salicin for 30 min. Reducing sugars released during hydrolysis were quantified using the 3,5-dinitrosalicylic acid (DNS) method by measuring the absorbance at 540 nm against a glucose standard curve (Wood and Bhat, 1988). The crude enzyme solution was heat-inactivated at 100 °C for 10 min and served as a reagent blank. One enzyme activity unit (U) was defined as the quantity of enzyme liberating 1 μmol of glucose equivalent per minute under the conditions described in Equation 1.


$$\mathrm{Enzyme}\,\mathrm{activity}\,\left(U/\mathrm{mL}\right)=\frac{\Delta \,C\times V_{t}\times 1000}{M\times V_{e}\times t}$$ (1)

where Δ*C* is the glucose concentration difference between the sample and blank (mg/mL), *V*_*t*_ is the total reaction volume (mL), *M* is the molecular weight of glucose (180.16 mg/mmol), *V*_*e*_ is the volume of the crude enzyme solution (mL) and t is the reaction time (min).

#### 2.2.2 Physiological and biochemical identification

Taxonomic identification was carried out on the automated GEN III MicroStation™ system. Cell suspensions adjusted to 90%–95% transmittance were dispensed into GEN III MicroPlates™ in line with the supplier’s protocol and the loaded plates were read automatically after 24 h of incubation at 28 °C (Liu et al., 2025). The resulting substrate-utilisation fingerprints were compared with the reference library embedded in the instrument (Bochner, 2009).

#### 2.2.3 Determination of straw degradation ratio and microstructural characterisation

Corn straw was air-dried, cut into 1–2 cm segments and dried at 60 °C to a constant weight to record the initial dry mass (*W*_0_), followed by sterilisation at 121 °C for 30 min. Aliquots of sterilised straw (20 g) were transferred into 1 L rectangular vessels. The moisture content was adjusted to 65% (w/w) with sterile water. Straw was inoculated at three cell densities (10^7^, 10^8^, and 10^9^ CFU/g), with uninoculated preparations serving as the control; all preparations were then held statically under aseptic conditions at 30 °C for 10 and 20 days. Residues were subsequently recovered by filtration through four gauze layers, dried at 60 °C to a constant weight (*W*_1_) and the degradation ratio was computed as Equation 2:


$$\mathrm{Degradation}\,\mathrm{ratio}=\frac{\left(W_{0}-W_{1}\right)}{W_{0}}\times 100\%$$ (2)

Straw samples were fixed with 2.5% glutaraldehyde in 0.1 mol/L phosphate buffer (pH 7.4) at 4 °C overnight, dehydrated through a graded ethanol series (30%–100%) and dried by critical point drying prior to gold sputter coating and observation (Duan et al., 2025; Wang et al., 2025).

### 2.3 Preliminary molecular identification based on 16S rRNA gene sequencing

Genomic DNA was extracted from the overnight cultures of each isolate using a Bacterial Genomic DNA Extraction Kit (Catalogue No. DP302; Tiangen Biochemical Technology Co., Ltd., China), according to the manufacturer’s instructions. The nearly full-length 16S rRNA gene (∼1,500 bp) was amplified by PCR using universal primers 27F (5′-AGAGTTTGATCCTGGCTCAG-3′) and 1492R (5′-TACGGCTACCTTGTTACGACTT-3′) (Weisburg et al., 1991). PCR was performed in a Bio-Rad T100 thermal cycler (Bio-Rad, USA) as follows: initial denaturation at 94 °C for 5 min; 35 cycles of denaturation at 94 °C for 30 s, annealing at 55 °C for 45 s and extension at 72 °C for 2 min, followed by a final extension at 72 °C for 10 min. Amplification products (1,500 bp) were verified by 1% (w/v) agarose gel electrophoresis and submitted to GenScript Biotechnology Co., Ltd. (Nanjing, China) for Sanger sequencing. The obtained sequences were queried against the NCBI 16S rRNA gene sequence database using the BLASTn algorithm. A phylogenetic tree was constructed using the neighbour-joining method with the Maximum Composite Likelihood model in MEGA 11 (Saitou and Nei, 1987; Tamura et al., 2021), with bootstrap support evaluated from 1,000 replicates. Reference sequences of closely related type strains were retrieved from the NCBI GenBank database.

### 2.4 Whole genome sequencing and assembly

Whole-genome sequencing was performed by Guangzhou Genedenovo Biotechnology Co., Ltd. (Guangzhou, China) using a combined long and short read strategy. PacBio continuous long reads (SMRTbell library) were used for *de novo* assembly and Illumina PE150 paired-end reads were used for consensus polishing. Long reads were used to obtain a closed chromosome, allowing the nine near-identical *rrn* operons and the horizontally acquired regions to be resolved and assigned unambiguous coordinates. Illumina raw reads were quality filtered with fastp v0.20.0, yielding 1.20 Gb of clean data (approximately 291 × coverage; Q30 91.6%). PacBio sequencing generated 2.67 Gb of subread data (198,462 reads; mean length 13.4 kb; read N50 15.2 kb; approximately 647 × coverage). The long reads were assembled *de novo* with Flye and the resulting assembly was polished with the Illumina short reads using Pilon v1.23 (Walker et al., 2014).

Assembly circularity was verified by joining the terminal sequences and remapping raw reads across the junction. Protein-coding genes were called with GeneMarkS v4.17 and functionally assigned by alignment to the KEGG and COG databases (Kanehisa et al., 2004; Galperin et al., 2015), while CAZyme content was annotated by BLASTP (E-value < 1e-5) against the CAZy database (Lombard et al., 2014), each gene being assigned the family label(s) of its best-scoring subject sequence. As a subject sequence may carry more than one family label, two counts are distinguished throughout: non-redundant CAZyme-encoding genes (distinct genes with at least one CAZy assignment) and gene family assignments (the sum of the family-level counts). Repetitive elements were resolved in two steps—interspersed repeats with RepeatMasker v4.0.5 and tandem repeats with Tandem Repeats Finder (v4.09). Non-coding RNAs were characterised by combining tRNAscan-SE (tRNAs), RNAmmer v1.2 (rRNAs) and cmscan v1.1.2 searches against the Rfam database for small regulatory RNAs (Lagesen et al., 2007). Genomic islands were predicted with IslandViewer 4, which integrates IslandPick, SIGI-HMM, IslandPath-DIMOB and Islander; prophage regions were identified with Phage_Finder v2.0 and CRISPR arrays were surveyed with CRISPRfinder v4.2.17 under default parameters.

Whole-genome assemblies of LSZ23 and its phylogenetic relatives were retrieved from GenBank. Phylogenomic analysis was conducted using REALPHY based on multiple reference genome alignments and the final tree was visualised and polished using iTOL v6 (Bertels et al., 2014; Letunic and Bork, 2024). For genomic classification, average nucleotide identity (ANI) was calculated with OrthoANI (v0.93.1; Chun et al., 2015).

### 2.5 Comparative genomic analysis

The genome of strain LSZ23 was compared with those of four reference strains: *B. subtilis* 168 (NZ_OZ024942.1), *Acetivibrio thermocellus* DSM 1313 (NC_017304.1), *B. velezensis* FZB42 (NC_009725.2), and *B. velezensis* SSF6 (GCF_030444515.1). *B. subtilis* 168 and the two *B. velezensis* strains were selected as phylogenetically close relatives within the *B. subtilis* species complex, whereas *A. thermocellus* DSM 1313, although phylogenetically distant, was included as a functional benchmark of high-efficiency cellulolysis rather than as a phylogenetic comparator. This panel does not include a *B. siamensis* genome; gene content identified below as absent from the reference set is therefore defined relative to these four genomes only and does not constitute a claim of uniqueness to strain LSZ23. All reference genome sequences were retrieved from the NCBI Genome database.

Orthologous gene families were identified across all five strains using OrthoFinder v2.5.4 (Emms and Kelly, 2019). Orthogroups were classified as core, accessory, or present in LSZ23 but absent from all four reference genomes. Functional annotation of the proteins encoded by the latter group was performed using InterProScan v5.62 (Jones et al., 2014) against the Pfam, TIGRFAM and CDD databases. The whole-genome collinearity between LSZ23 and each reference strain was assessed using MUMmer v3.23 (Kurtz et al., 2004) and visualised using TBtools v1 (Chen et al., 2020).

### 2.6 Differential expression analysis

Two culture conditions were compared, each with three biological replicates: a straw-treatment group, in which LSZ23 was cultivated in LB medium supplemented with 2% (w/v) sterilised corn straw and a control group (CK) in LB medium without straw, *n* = 3 per group. Both groups were inoculated with 3% (v/v) of an overnight culture and incubated at 30 °C and 170 rpm. At 120 h, the time point at which CMCase activity reached its maximum during a preliminary 6-day enzyme monitoring (Supplementary Figure 4), cultures were harvested by centrifugation at 12,000 × *g* for 5 min at 4 °C. The cell-free supernatant was retained for determination of cellulase activities and the cell pellet was used for total RNA and intracellular metabolite extraction. Total RNA was extracted using a Bacterial RNA Rapid Extraction Kit (Spark Jade, China) according to the manufacturer’s instructions and RNA quality and concentration were assessed using a NanoDrop 2000 spectrophotometer (Thermo Fisher Scientific, USA). Transcriptomic sequencing was performed on the NovaSeq X Plus (Illumina) and DNBSEQ-T7 (MGI) platforms, with three biological replicates per condition.

Raw reads were quality filtered with fastp v0.23.4 (Chen et al., 2018) to remove adapter sequences and low-quality bases, yielding 19.26 Gb of clean data in total (≥2.99 Gb per sample; Q30 ≥ 96.66%). Clean reads were aligned to the *B. siamensis* LSZ23 chromosome (GenBank accession CP113001) with Bowtie2 v2.5.4 (Langmead and Salzberg, 2012). Per-gene expected counts and TPM values were generated with RSEM v1.3.3 (Li and Dewey, 2011). Differentially expressed genes (DEGs) were identified using the DESeq2 R package v1.42.0 (Love et al., 2014) and defined as genes with —log_2_(fold change)| > 1 and a Benjamini–Hochberg adjusted *P*-value (*P*_*adj*_) < 0.05. The DEGs were further screened for CAZyme-encoding genes based on CAZy database annotations. KEGG pathway enrichment analysis was performed to identify the key genes and pathways associated with carbohydrate metabolism and cellulose degradation and downstream visualisation was conducted using the Majorbio Cloud Platform.^1^

### 2.7 Metabolomics analysis

For metabolomic profiling, six biological replicates per condition were harvested at 120 h from LB medium with or without 2% (w/v) sterilised corn straw, incubated at 30 °C and 170 rpm (see section “2.6 Differential expression analysis” for full culture details). Each bacterial pellet (50 mg) was extracted in 400 μL of pre-chilled methanol–water (4:1, v/v). The mixture was homogenised using a high-throughput tissue disruptor (Wonbio-96c, Shanghai Wanbo Biotechnology Co., Ltd.) at low temperature, vortexed and ultrasonicated on ice for 30 min. After incubation at −20 °C for 30 min, samples were centrifuged at 13,000 × *g* for 15 min at 4 °C and the resulting supernatants were transferred to LC-MS vials for analysis. Metabolite profiling was performed using a UHPLC-Q Exactive HF-X system (Thermo Fisher Scientific, Waltham, MA, USA) operating in both positive and negative ionisation modes. Raw data were processed and metabolites were identified by database matching against the HMDB, KEGG and Metlin databases. Significantly DAMs were identified as features with a variable importance in projection (VIP) value > 1.0, derived from partial least squares discriminant analysis (PLS-DA), together with a Student’s *t*-test *P*-value adjusted by the Benjamini–Hochberg procedure (*P*_*adj*_ < 0.05). Model reliability was validated by 200 response permutation testing; a model was considered valid when the permuted R^2^Y and Q^2^ were lower than the original and the Q^2^ regression intercept was negative. Downstream statistical analyses and visualisations were performed using the Majorbio Cloud Platform (Westerhuis et al., 2008; Fang et al., 2025).

To identify pathways co-regulated at the transcriptional and metabolic levels, KEGG pathway enrichment results from the DEGs and DAMs were integrated. A pathway was regarded as jointly responsive when it was enriched in both datasets and its combined significance was evaluated from the transcriptomic and metabolomic enrichment *P*-values. Pathways reaching *P* < 0.05 in both layers were considered jointly significant. A correlation network was constructed using the three biological replicates per group for which transcriptomic, metabolomic and CMCase activity measurements were all available (*n* = 6 in total); metabolite abundances for these matched cultures were taken from the corresponding replicates of the metabolomics dataset. CMCase activity was determined for each of these matched cultures as described above. Network construction and visualisation were performed using R software v4.5.2, where Pearson correlation coefficients were calculated and the correlation circle network was rendered using base R graphics with the readxl and svglite packages.

### 2.8 Statistical analyses

Statistical analyses were performed in IBM SPSS Statistics v26.0. Differences among more than two groups were assessed by one-way analysis of variance (ANOVA) followed by Fisher’s least significant difference (LSD) test and differences between the straw and CK groups by independent-samples *t*-test, *P* < 0.05 was considered significant. Multiple testing was accounted for using the Benjamini–Hochberg procedure, applied to the differential gene expression analysis, to the GO and KEGG enrichment analyses and to the differential metabolite analysis; adjusted values are reported throughout as *P*_*adj*_. *P*-values reported for Pearson correlations and for the ANOVA-based comparisons are unadjusted. Statistical procedures for the transcriptomic and metabolomic data are described in sections “2.6 Differential expression analysis” and “2.7 MetabolomicsAnalysis.” Graphs were prepared in GraphPad Prism v9.0.0 and OriginPro v2025.

## 3 Results

### 3.1 Isolation and screening of lignocellulose-degrading bacteria

Four bacterial strains exhibiting lignocellulolytic activity, designated BX3, BX11, BX15, and LSZ23, were isolated from humic soil and further screened on three media, targeting distinct lignocellulose fractions: carboxymethyl cellulose, microcrystalline cellulose and aniline blue. Across all three substrates, strain LSZ23 generated clear hydrolysis halos around its colonies and exhibited significantly greater hydrolytic capacity than the remaining three isolates (*P* < 0.05; Supplementary Table 1), with mean HCR values of 4.24 ± 0.11, 4.97 ± 0.06, and 1.80 ± 0.04 (Figure 1A, a–c). The pronounced haloes on both the amorphous and the crystalline cellulose substrates indicated that LSZ23 possessed a balanced cellulolytic repertoire. LSZ23 was therefore prioritised as the most promising candidate for further characterisation.

**FIGURE 1:**
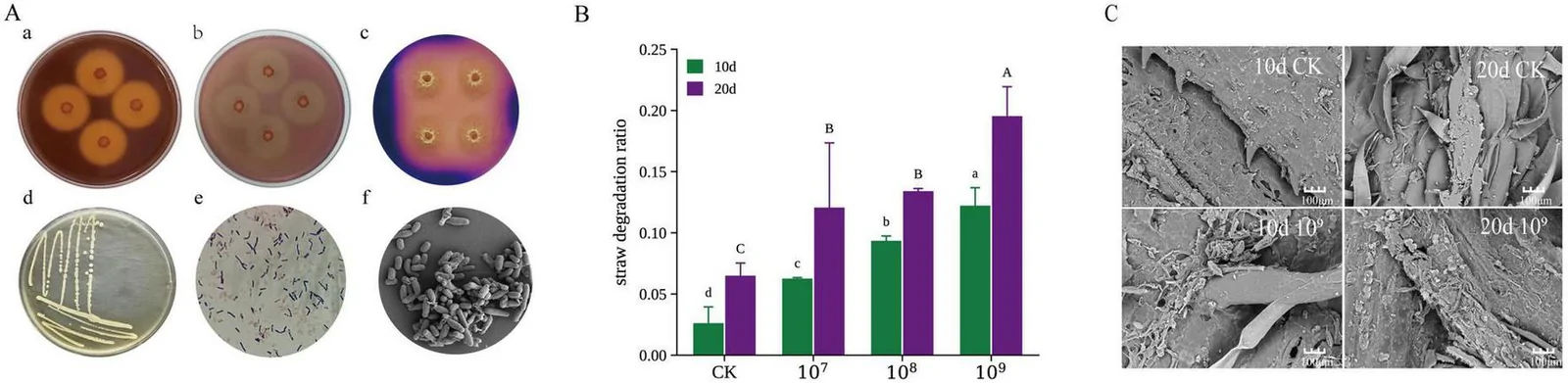
Morphological characterisation and degradation performance of strain LSZ23. **(A)** Qualitative screening for hydrolytic activities of strain LSZ23 on different agar media. (a–c) Hydrolysis zones on carboxymethyl cellulose (CMC), Avicel and Aniline blue plates, respectively; (d) Colony morphology on Luria–Bertani (LB) agar; (e) Gram staining observation under a light microscope; (f) Scanning electron microscopy (SEM) image showing the rod-shaped cell morphology. **(B)** Straw degradation ratio of strain LSZ23 at different inoculation concentrations (10^7^, 10^8^, and 10^9^ colony-forming units (CFU)/*g*) after 10 and 20 days of incubation. Data are presented as mean ± standard deviation of three biological replicates. Different lowercase letters above the 10 days bars and different uppercase letters above the 20 days bars indicate significant differences among treatments at the respective sampling time (one-way ANOVA followed by Fisher’s LSD test; *P* < 0.05). **(C)** SEM observation of straw surface microstructure. SEM structural analysis revealed that the cell wall lattice of corn straw remained highly ordered and intact in the control groups (CK) at both 10 and 20 days. In contrast, LSZ23-treated substrates exhibited microvacuolisation, extensive fibre bundle delamination and pronounced surface pitting, confirming active structural deconstruction.

### 3.2 Physiological and biochemical characteristics

Morphological identification demonstrated that LSZ23 formed smooth, wrinkled grey colonies and grew aerobically; cells were Gram-positive, rod-shaped and spore-forming under light microscopy (Figure 1A, d–f). Crude enzyme assays showed that LSZ23 produced all four canonical cellulolytic activities: CMCase (0.321 ± 0.006 U/mL), β-glucosidase (0.275 ± 0.001 U/mL), avicelase (0.240 ± 0.008 U/mL), and FPase (0.209 ± 0.004 U/mL) (Supplementary Table 2). The co-occurrence of endo-acting (CMCase), exo-acting (avicelase) and cellobiose-hydrolysing (β-glucosidase) activities indicated a functionally balanced hydrolytic apparatus capable of cooperative cellulose depolymerisation, in which β-glucosidase activity mitigates the cellobiose-driven feedback inhibition acting on the upstream cellulases.

Physiological and biochemical profiles obtained using the GEN III MicroStation system revealed 25 positive reactions. Strain LSZ23 was able to utilise six carbohydrate substrates for growth, including dextrin, gentiobiose, sucrose, α-D-glucose, D-mannose, and D-fructose, as well as three amino acid substrates (L-alanine, L-aspartic acid, and D-aspartic acid). Positive responses were also observed towards four chemical agents: sodium lactate, tetrazolium violet, sodium butyrate and sodium bromate. In addition, LSZ23 was susceptible to five antimicrobials: rifamycin SV, minocycline, erythromycin, lincomycin, and vancomycin (Supplementary Figure 1).

In the straw degradation assay, LSZ23 depolymerised the corn straw in a concentration- and time-dependent manner (Figure 1B). At 10 days, inoculation at 10^7^, 10^8^, and 10^9^ CFU/g produced degradation ratios of 6.25 ± 0.09%, 9.35 ± 0.39%, and 12.22 ± 1.47%, respectively, each significantly greater than the uninoculated CK (2.61 ± 1.34%; *P* < 0.05) and significantly different from one another. By 20 days, the corresponding ratios were 12.06 ± 5.29%, 13.40 ± 0.22%, and 19.54 ± 2.41%, compared with 6.49 ± 1.04% for the CK. The degradation ratio at 10^9^ CFU/g was significantly higher than that in all other treatments at this time point (*P* < 0.05), whereas the 10^7^ and 10^8^ groups did not differ significantly from each other. The 10^9^ CFU/g treatment, which yielded the highest degradation ratio among the densities tested, was used for the subsequent microstructural characterisation. At the same time point, scanning electron microscopy corroborated the degradation at the microstructural level (Figure 1C); control surfaces remained smooth with intact, well-defined fibre bundles, whereas LSZ23-treated straw (10^9^ CFU/g) showed extensive disruption, pronounced porosity, crack formation and cavity development, consistent with active enzymatic attack on the lignocellulosic matrix.

### 3.3 Taxonomic identification based on 16S rRNA and whole genome phylogenetic analyses

The amplified 16S rRNA gene sequence of LSZ23 was approximately 1,500 bp, matching the expected size. Phylogenetic analysis revealed that strain LSZ23 is closely related to members of the genus Bacillus, forming a highly supported clade (99% bootstrap value) with the type strains *B. amyloliquefaciens* MPA 1034^T^ and *B. siamensis* KCTC 13613^T^ (Supplementary Figure 2). Based on its morphological characteristics, physiological–biochemical properties and 16S rRNA gene sequence analysis, strain LSZ23 was identified as a member of the genus *Bacillus*. However, owing to the high conservation of 16S rRNA gene sequences among members of this complex (>99% interspecies identity), single-gene phylogeny is insufficient for reliable species-level resolution; therefore, whole-genome-based analyses were pursued. The species-level identification presented below therefore rests on core-genome phylogeny and ANI, with the 16S rRNA analysis serving only to support the genus-level assignment.

Whole-genome sequencing revealed that LSZ23 possessed a single circular chromosome of 4.12 Mb with a GC content of 46%, encoding 4,120 protein-coding genes (Figure 2A). No plasmid was detected. The chromosome harboured nine complete *rrn* operons (27 rRNA genes: nine 16S, nine 23S, and nine 5S), together with 87 tRNA genes. Dispersed repetitive content was modest: 77 interspersed repeat elements (5,303 bp; 0.13% of the chromosome) and 116 tandem repeats (10,965 bp; 0.27% of the chromosome). No CRISPR array was detected. In contrast, horizontally acquired content was substantial: 11 genomic islands spanning 298,810 bp (mean 27.2 kb; 7.26% of the chromosome) and seven prophage regions spanning 266,703 bp (mean 38.1 kb; 6.48%), each between 24.6 and 51.0 kb and all classified as large prophages. Prophage and genomic island predictions partially overlapped and the two categories are therefore not additive. This closed, chromosome-level assembly provided the basis for the functional and comparative genomic analyses described below. A comparison of all annotated proteins against the NCBI Nr database demonstrated that the largest fraction of LSZ23 genes (2,238 genes) showed the greatest sequence similarity to *B. siamensis*, substantially exceeding the number assigned to any other species (Figure 2B).

**FIGURE 2:**
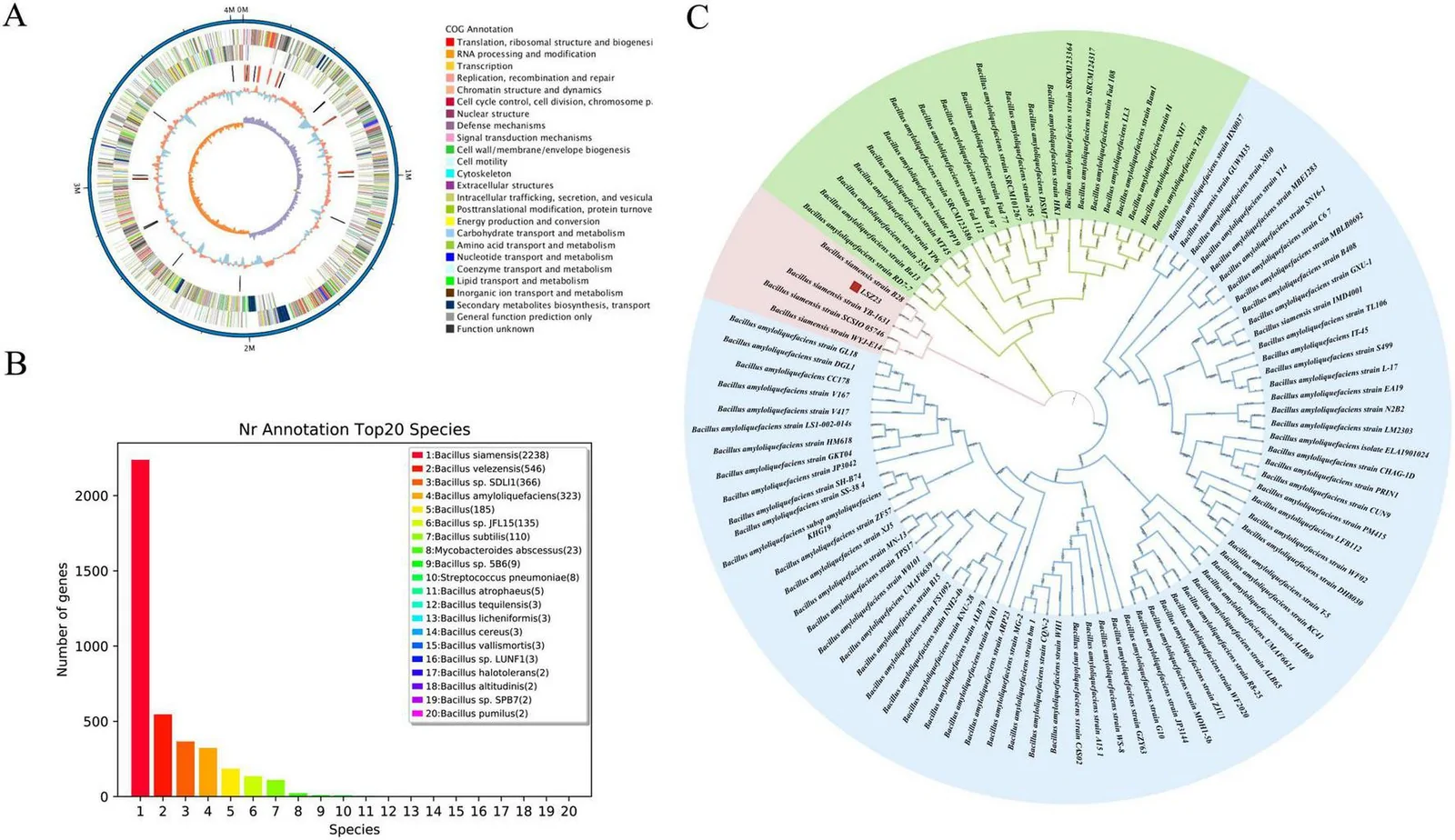
Genomic characteristics and phylogenetic analysis of strain LSZ23. **(A)** Circular genome map of strain LSZ23. From the outside to the inside: the first and fourth circles represent the COG (Clusters of Orthologous Groups) functional classification of genes on the forward and reverse strands, respectively; the second and third circles show the genes on the forward and reverse strands; the fifth circle displays the GC content (black); and the sixth circle indicates the GC skew (green/purple). **(B)** Distribution of top 20 species based on Nr (non-redundant protein) database annotation. The bar chart shows the number of genes assigned to different species, with *Bacillus siamensis* showing the highest homology to strain LSZ23. **(C)** Phylogenetic tree of strain LSZ23 and related species. The circular phylogenetic tree was constructed based on genomic sequences using the Maximum Likelihood (ML) method implemented in REALPHY. Strain LSZ23 is highlighted (red box) and clusters closely with *B. siamensis* and *Bacillus velezensis* strains, consistent with the average nucleotide identity (OrthoANI) results.

To ascertain the taxonomic status and evolutionary trajectory of strain LSZ23, a whole-genome phylogenetic reconstruction was conducted using core-genome sequences from 104 representative *Bacillus* strains. The phylogenetic tree topology resolved distinct monophyletic clades, clearly separating the *B. siamensis* lineage from the broader *B. amyloliquefaciens* species complex (Figure 2C). Strain LSZ23 clustered robustly within the *B. siamensis* clade, exhibiting the highest genetic affinity to *B. siamensis* B28, as supported by high bootstrap confidence. This distinct phylogenetic placement suggests a divergent evolutionary path from other common *Bacillus* species, potentially underlying its specific metabolic adaptations. Furthermore, OrthoANI analysis of LSZ23 and nine closely related *Bacillus* type strains confirmed a pairwise identity of 99.65% with *B. siamensis* B28 (Supplementary Table 3). This value lies well above the 95%–96% ANI cut-off conventionally applied to delimit prokaryotic species. Collectively, the core genome topology and OrthoANI values unambiguously classified the strain LSZ23 as *B. siamensis*.

### 3.4 Genomic structural characteristics

COG functional annotation assigned 2,853 of the 4,120 predicted proteins to functional categories (Figure 3A). Excluding poorly informative “General function prediction only (R)” and “Function unknown (S)” categories, the three most abundant categories were amino acid transport and metabolism (342 genes, 12.0%), transcription (294 genes, 10.3%) and carbohydrate transport and metabolism (234 genes, 8.2%). Within the carbohydrate transport and metabolism category, the most prevalent COG families were COG2814 (predicted arabinose efflux permease AraJ, MFS family), COG0726 (peptidoglycan/xylan/chitin deacetylase, PgdA/CDA1 family), COG1349 (DNA-binding transcriptional regulator of sugar metabolism, DeoR/GlpR family) and COG0697 (permease of the drug/metabolite transporter superfamily), collectively reflecting the genomic capacity for diverse carbohydrate sensing, transport and catabolism. KEGG pathway annotation mapped 1,207 genes to metabolic pathways (Figure 3B). Within the carbohydrate metabolism category (229 genes), the most gene-rich pathways were pyruvate metabolism (ko00620; 42 genes), glycolysis/gluconeogenesis (ko00010; 37 genes), amino sugar and nucleotide sugar metabolism (ko00520; 37 genes), glyoxylate and dicarboxylate metabolism (ko00630; 34 genes), propanoate metabolism (ko00640; 34 genes) and starch and sucrose metabolism (ko00500; 33 genes) (Supplementary Table 4).

**FIGURE 3:**
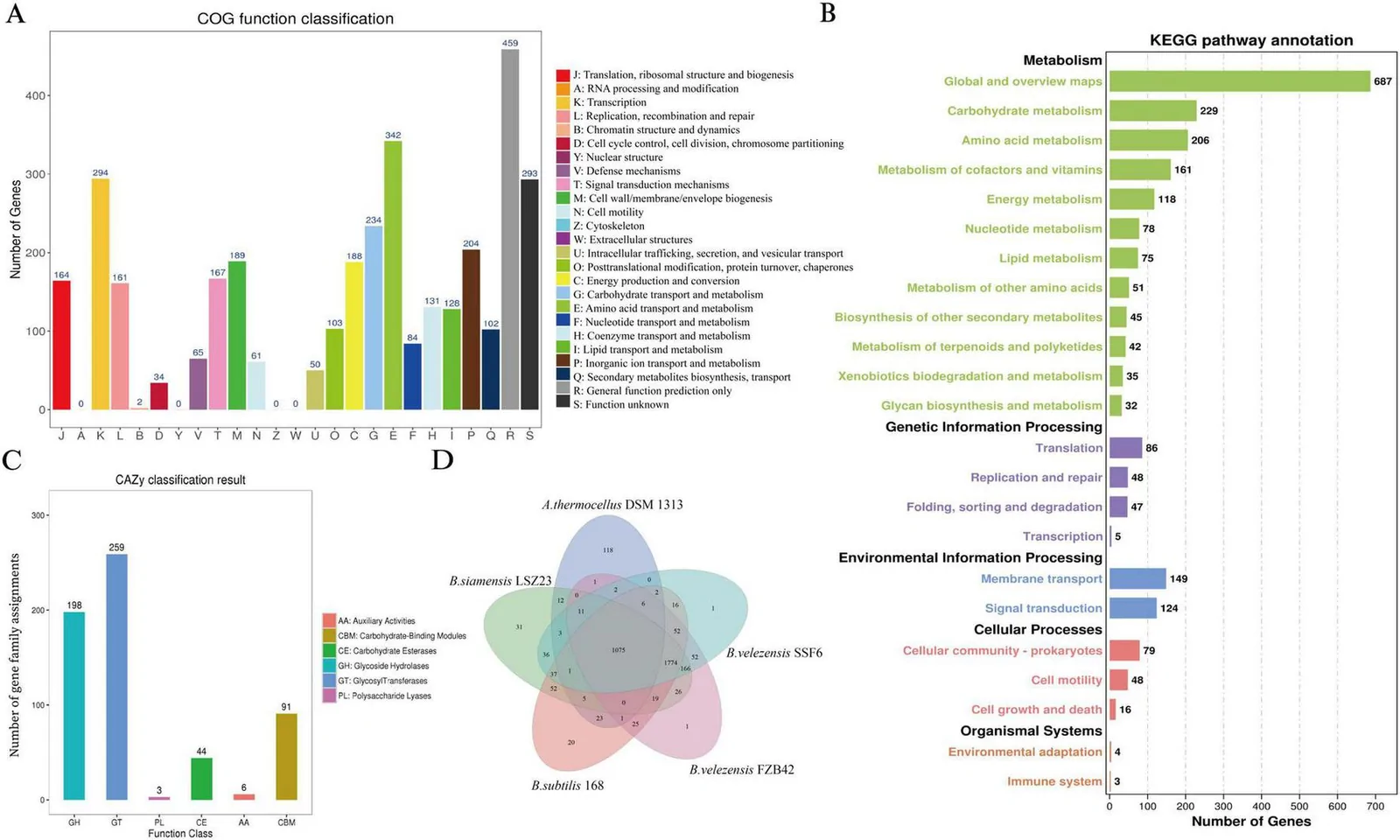
Functional classification and annotation of the strain LSZ23 genome. **(A)** Clusters of Orthologous Groups (COG) functional classification. The bar chart illustrates the distribution of genes into various COG categories. The categories related to “Carbohydrate transport and metabolism” (G), “General function prediction only” (R) and “Amino acid transport and metabolism” (E) are prominently represented. **(B)** Kyoto Encyclopedia of Genes and Genomes (KEGG) pathway annotation. The horizontal bar chart shows the enrichment of genes across different KEGG functional hierarchies. The “Metabolism” category, particularly “Carbohydrate metabolism,” and “Amino acid metabolism,” contains the highest number of annotated genes. **(C)** Carbohydrate-active enzyme (CAZy) classification results. Bars show the number of gene family assignments per CAZy class. Glycoside Hydrolases (GH), Glycosyltransferases (GT), Polysaccharide Lyases (PL), Carbohydrate Esterases (CE), Auxiliary Activities (AA), and Carbohydrate-Binding Modules (CBM). A gene whose best-scoring subject sequence carries more than one CAZy family label contributes one assignment to each class concerned. Twelve genes within the GH class are assigned to the unclassified GH0 category. **(D)** Venn diagram of orthogroup distribution among the five bacterial strains compared, showing shared orthogroups and those detected in only one of the five genomes. The diagram illustrates the distribution of orthogroups identified by OrthoFinder across *Acetivibrio thermocellus* DSM 1313, *Bacillus siamensis* LSZ23, *Bacillus subtilis* 168, *Bacillus velezensis* FZB42, and *B. velezensis* SSF6.

Carbohydrate-active enzyme annotation identified 570 predicted non-redundant CAZyme-encoding genes. Because 26 of these genes have a best-scoring subject sequence carrying two or more CAZy family labels, those 26 genes contribute 57 family assignments, so that the 570 genes account in total. At the class level these comprise 198 glycoside hydrolase (GH), 259 glycosyltransferase (GT), 91 carbohydrate-binding module (CBM), 44 carbohydrate esterase (CE), six auxiliary activity (AA), and three polysaccharide lyase (PL) assignments (Figure 3C and Supplementary Table 5). The 198 GH assignments correspond to 41 GH families once subfamilies are merged, together with 12 genes assigned to the unclassified GH0 category (55 family and subfamily rows in Supplementary Table 5). Merging subfamilies, the most abundant GH families were GH13 (27), GH1 (19), GH23 (19), GH32 (12), and GH73 (11). Key cellulolytic GH families included GH5 (endoglucanases, three genes; one carrying a CBM3 cellulose-binding module), GH1 and GH3 (β-glucosidases, 27 genes combined), which collectively constitute the core cellulose degradation machinery. The 259 GT assignments were dominated by GT2 (112) and GT4 (69), which are involved in cell wall and polysaccharide biosynthesis, respectively. CBMs (91) spanned 15 families, with CBM50 (56), CBM48 (7), and CBM5 (7) being the most abundant; CBM50 mediates peptidoglycan binding, whereas CBM5 and CBM48 facilitate substrate binding during polysaccharide degradation. CEs (44) were assigned to eight families, including CE4 (14), CE14 (10), CE1 (6), and CE9 (5), which remove acetyl and other substituents from the polysaccharides. Three PL genes were identified, representing two families: PL1 and PL9. Finally, the six AA assignments fell into four families: AA1 (2), AA7 (2), AA10 (1), and AA4 (1).

### 3.5 Comparative genomic analysis

To contextualise the genomic features of LSZ23 within a broader evolutionary and functional framework, its genome was compared with those of four reference strains: *B. subtilis* 168 (NZ_OZ024942.1), *B. velezensis* FZB42 (NC_009725.2), *B. velezensis* SSF6 (GCF_030444515.1) and *A. thermocellus* DSM 1313 (NC_017304.1). The latter was included as a representative benchmark of high-efficiency cellulose degradation (Supplementary Table 6). The genome of LSZ23 spanned 4.12 Mb with 4,120 predicted coding sequences (CDS) and a GC content of 46%, values comparable to those of *B. velezensis* FZB42 (3.92 Mb; 3,736 CDS; 46% GC) and SSF6 (3.89 Mb; 3,717 CDS; 47% GC), consistent with the conserved genomic background of the *B. subtilis* species complex. *B. subtilis* 168 carried a slightly larger genome (4.22 Mb; 4,226 CDS) with a lower GC content (44%), whereas the obligate anaerobe *A. thermocellus* DSM 1313 exhibited a markedly reduced genome size (3.56 Mb), fewer coding genes (3,068) and a substantially lower GC content (39%), consistent with its phylogenetic distance and anaerobic lifestyle.

The whole-genome collinearity analysis revealed extensive chromosomal synteny between LSZ23 and both *B. velezensis* strains (FZB42 and SSF6), with continuous collinear blocks spanning nearly the entire genome (Supplementary Figure 3), indicating a recent common ancestry and conserved genome organisation within this clade. Comparison with *B. subtilis* 168 revealed substantial but fragmented synteny punctuated by multiple large-scale chromosomal rearrangements and localised inversions, reflecting divergent evolution within the genus *Bacillus*. As anticipated, given their deep phylogenetic separation, the collinearity between LSZ23 and *A. thermocellus* DSM 1313 was minimal and largely limited to conserved housekeeping gene regions.

Pan-genomic analysis using OrthoFinder delineated shared and strain-restricted gene repertoires across all five strains (Figure 3D). A total of 1,075 orthogroups were shared among all five genomes, constituting the core genome and encompassing fundamental cellular functions, including DNA replication, transcription and translation. A total of 1,774 orthogroups were shared among LSZ23 and the three *Bacillus* references but absent from *A. thermocellus*, further underscoring its phylogenetic affinity within the *B. subtilis* complex. Notably, LSZ23 harboured 31 orthogroups (OGs) that were absent from all four reference genomes, encoding 92 proteins (Supplementary Table 7). InterProScan annotation assigned putative functions to 68 of these 92 proteins (73.9%), including ABC transporters (*n* = 5), DNA modification-related proteins (*n* = 5), signal transduction components (*n* = 4) and transcriptional regulators (*n* = 2), together with a set of carbohydrate-active proteins comprising two GH13-family α-amylases, one GH65-family glycoside hydrolase and a CBM26 starch-binding module.

### 3.6 Transcriptomic response of LSZ23 to corn straw substrate

At 120 h (Supplementary Figure 4), total RNA was isolated from LSZ23 grown in LB supplemented with 2% straw (straw group) and in LB without straw (the CK group). Differential expression analysis yielded 173 DEGs (—log_2_FC| > 1, *P*_*adj*_ < 0.05), of which 85 were upregulated and 88 were downregulated (Figure 4A). Of these, 104 were assigned KEGG orthology annotations and 46 were annotated in the Gene Ontology (GO) database.

**FIGURE 4:**
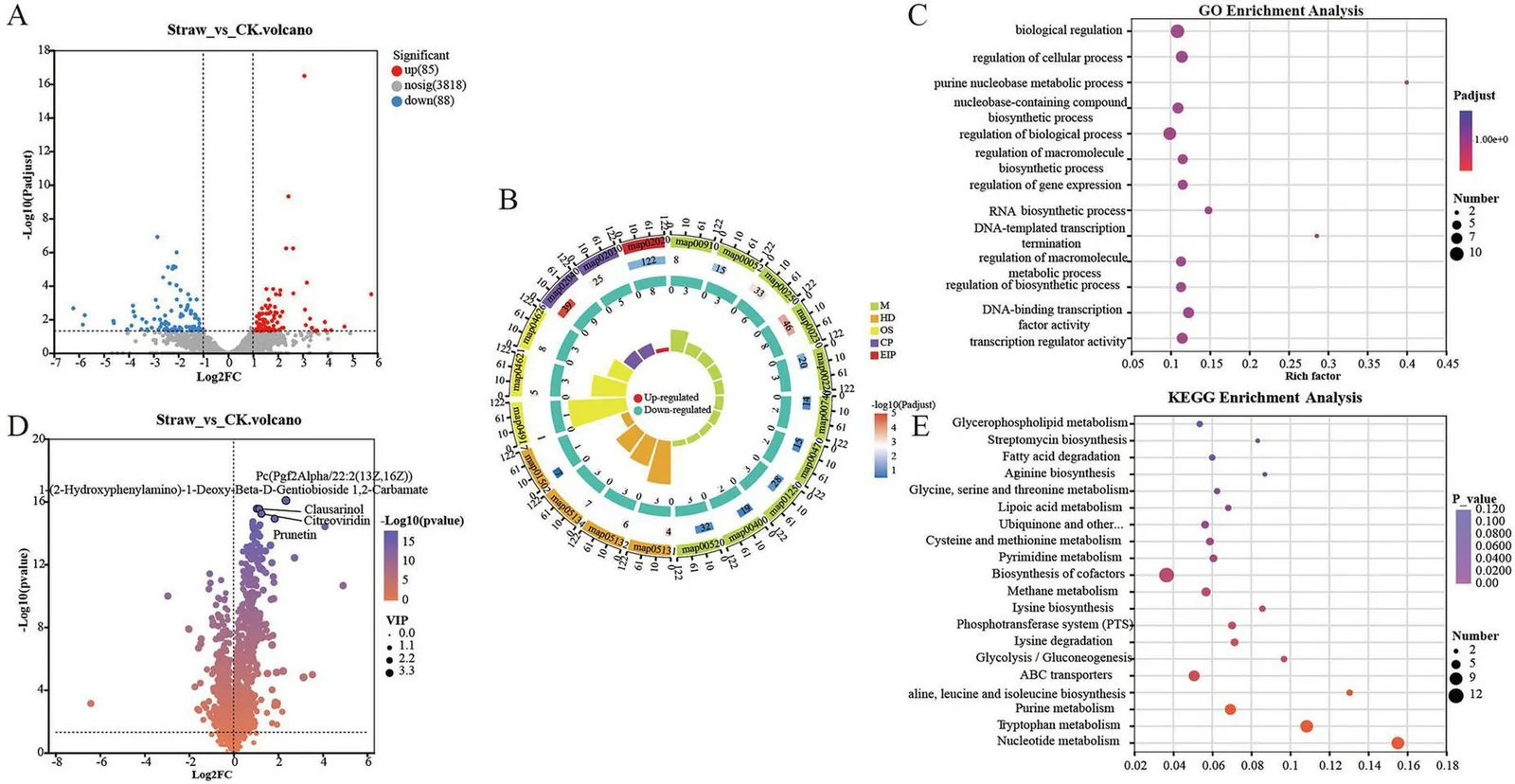
Integrated transcriptomic and metabolomic analysis of strain LSZ23. **(A)** Volcano plot of differentially expressed genes (DEGs) between straw and the control (CK, LB). The red and blue dots represent significantly upregulated and downregulated genes, respectively (—log_2_ fold change (log_2_FC)| > 1 and Benjamini–Hochberg *P*_*adj*_ < 0.05), while grey dots indicate genes with no significant change. **(B)** Circular visualisation of DEG enrichment across significantly changed Kyoto Encyclopedia of Genes and Genomes (KEGG) pathways. The inner rings represent the upregulated (red) and downregulated (green) genes associated with specific metabolic functions. **(C)** Gene Ontology (GO) enrichment analysis of DEGs (*P*_*adj*_ < 0.05). The bubble plot displays the top GO terms enriched in the transcriptomic data. The size of the bubbles represents the number of genes and the colour indicates the significance level. **(D)** Volcano plot illustrating differentially abundant metabolites (DAMs, *P*_*adj*_ < 0.05, independent-samples *t*-test with Benjamini–Hochberg correction, *P*_*adj*_ < 0.05) between the straw treatment and the control (CK). This visualisation characterises significantly altered metabolites based on log_2_ fold change (log_2_FC) and variable importance in projection (VIP) values, with specific key metabolites such as choline and adenosine highlighted. **(E)** KEGG pathway enrichment analysis of the DAMs (independent-samples *t*-test with Benjamini–Hochberg correction). The bubble plot highlights the metabolic pathways that were most significantly affected in LSZ23.

KEGG pathway enrichment analysis identified 12 significantly enriched pathways (*P*_*adj*_ < 0.05; Figure 4B and Supplementary Table 8). Flagellar assembly (map02040) was the most enriched pathway, comprising 10 DEGs, followed by purine metabolism (map00230; 8 DEGs); alanine, aspartate and glutamate metabolism (map00250; 7 DEGs); bacterial chemotaxis (map02030; 5 DEGs); nitrogen metabolism (map00910; 4 DEGs); galactose metabolism (map00052; 4 DEGs); and arginine biosynthesis (map00220; 4 DEGs). Constituent DEGs were predominantly downregulated in the flagellar assembly and bacterial chemotaxis pathways. Three flagellin *hag* paralogues were downregulated (*P*_*adj*_ < 0.05), together with the genes encoding the flagellar hook-associated proteins (*flgK*, *flgL*), export chaperone *fliS*, regulatory protein *fliT* (*P*_*adj*_ < 0.05) and motor protein *motB*. Six genes encoding methyl-accepting chemotaxis proteins were downregulated (*P*_*adj*_ < 0.05). In contrast, the amyloid fibre anchoring and assembly protein gene *tapA* was significantly upregulated (*P*_*adj*_ < 0.05). DEGs related to purine metabolism were uniformly downregulated, spanning *de novo* biosynthesis (*purF* and *purC*) and xanthine salvage and transport (*xpt* and *pbuX*). In nitrogen metabolism, key components of the GS-GOGAT (glutamine synthetase-glutamate synthase) pathway were significantly suppressed; specifically, the glutamate synthase (GOGAT) subunits *gltD* and *gltB*, along with the NADP-specific glutamate dehydrogenase *gdhA*, were downregulated. Conversely, the pyruvate dehydrogenase E1α subunit *pdhA* and a COX15/CtaA-family cytochrome oxidase assembly protein gene were upregulated (*P*_*adj*_ < 0.05). Among the DEGs related to carbohydrate utilisation, the PTS lactose/cellobiose transporter subunit IIA gene and a GH1-family glucosidase gene were upregulated (*P*_*adj*_ < 0.05), whereas three galactose metabolism genes, *galT*, *galE* and a galactokinase gene, were downregulated (*P*_*adj*_ < 0.05). The xylulokinase gene was upregulated. Five MarR-family transcriptional regulator genes were upregulated (*P*_*adj*_ < 0.05; Supplementary Table 9).

Among the lignocellulose degradation genes detected in the transcriptome, most were transcribed but were not significantly differentially expressed between the straw and control conditions, indicating that only a defined subset responded to the straw (Supplementary Table 10). The induced subset converged on hemicellulose depolymerisation and cellobiose utilisation, a GH11 endo-1,4-β-xylanase (log_2_FC = 3.90, *P*_*adj*_ = 0.045) and a GH1 β-glucosidase (log_2_FC = 3.36, *P*_*adj*_ = 0.033) were significantly upregulated, as were the cellobiose-specific PTS transporter component (log_2_FC = 3.88, *P*_*adj*_ = 0.015) and xylulokinase (log_2_FC = 3.56, *P*_*adj*_ = 0.047). Consistently, the adjacent pentose catabolic and transport genes, including xylose isomerase (*xylA*), L-arabinose isomerase (*araA*) and a second cellobiose PTS permease (*celB*), showed the same upward trend (log_2_FC = 2.7–3.6), albeit below the significance threshold. Together, these results indicate that straw triggers a focused hemicellulose- and cellobiose-centred catabolic response in LSZ23, rather than a broad induction of cellulolytic enzymes.

Gene Ontology enrichment analysis of the 46 annotated DEGs identified 13 significantly enriched terms (*P*_*adj*_ < 0.05; Figure 4C and Supplementary Table 11). Within the Biological Process ontology, biological regulation (GO:0065007; 10 genes) and the regulation of cellular processes (GO:0050794; eight genes) were the most enriched terms. In the Molecular Function ontology, DNA-binding transcription factor activity (GO:0003700; seven genes) was the most significantly enriched term.

### 3.7 Metabolomic response of LSZ23 to corn straw substrate

To elucidate the metabolic adaptation of strain LSZ23 during lignocellulose degradation, the straw and CK metabolomes were profiled and contrasted. In an unsupervised PCA, the two conditions occupied clearly separated regions of the score plot and the leading three components together explained 80.0% of the total variance (PC1 = 60.9%, PC2 = 11.7%, PC3 = 7.4%; Supplementary Figure 5). This grouping was further confirmed using a supervised PLS-DA model, which exhibited robust explanatory and predictive reliabilities (R^2^Y = 0.994, Q^2^ = 0.947, Supplementary Figure 6). Based on stringent screening criteria (VIP > 1.0, *P*_*adj*_ < 0.05), pairwise comparison identified 1,094 significant DAMs, comprising 612 increased and 482 decreased features, in the straw group relative to CK (Figure 4D).

KEGG pathway enrichment analysis of the 1,094 DAMs identified 12 significantly enriched pathways (*P*_*adj*_ < 0.05; Figure 4E and Supplementary Table 8), including nucleotide metabolism; tryptophan metabolism; purine metabolism; valine, leucine and isoleucine biosynthesis; ABC transporters; glycolysis/gluconeogenesis; lysine degradation; phosphotransferase system; lysine biosynthesis; methane metabolism; biosynthesis of cofactors; and pyrimidine metabolism. The two most significantly enriched pathways, nucleotide and tryptophan metabolism, were characterised by predominantly downregulated constituent metabolites. Within tryptophan metabolism, the levels of indole-3-acetic acid (IAA), indole-3-acetamide and N-acetylserotonin were reduced in the straw group. Within central carbon metabolism, the glycolysis/gluconeogenesis pathway was significantly enriched. Of the three DAMs with KEGG annotation in this pathway, 3-phosphoglycerate and acetyl-CoA were classical glycolytic intermediates, whereas salicin, a glucoside transported via the PTS, was annotated and downregulated in this pathway (*P*_*adj*_ < 0.05).

In contrast, aromatic and phenolic compounds were extensively upregulated in the straw group (Supplementary Table 12). Three DAMs annotated in the KEGG “degradation of aromatic compounds” pathway (map01220) were significantly upregulated (*P*_*adj*_ < 0.05), namely 4-hydroxycinnamic acid, salicylic acid and p-anisic acid. An additional group of lignin-derived aromatic compounds and hydroxycinnamic acid derivatives was also significantly upregulated, including syringaldehyde, decarboxylated 8,5’-diferulic acid, 1,4-bis(4-hydroxy-3-methoxyphenyl)-2,3-dimethylbut-2-ene-1,4-dione, isoferulic acid, ethyl p-coumarate, 5-hydroxyferulate and vanillin (*P*_*adj*_ < 0.05). The biosynthesis of cofactors pathway was significantly enriched (*P*_*adj*_ < 0.05), with statistically significant increases in the abundances of nicotinic acid, quinolinic acid and β-nicotinamide adenine dinucleotide detected in the straw group. S-adenosylhomocysteine (SAH), an intermediate of the methyl cycle, was also upregulated. Additionally, the valine, leucine and isoleucine biosynthesis pathways was significantly enriched (*P*_*adj*_ = 0.012), with the upstream precursor 2-isopropylmalic acid showing the highest fold-change among the KEGG-annotated DAMs in this pathway.

### 3.8 Integrated multiomics analysis

To identify the co-regulated pathways governing the transcriptional and metabolic responses during corn straw degradation, KEGG enrichment results from the transcriptomic and metabolomic datasets were jointly analysed, yielding 19 co-enriched pathways (Figure 5A). Purine metabolism (map00230) was the only pathway that simultaneously reached statistical significance at both omics levels (transcriptomics: 8 DEGs, *P* < 0.001; metabolomics: 7 DAMs, *P* = 0.005).

**FIGURE 5:**
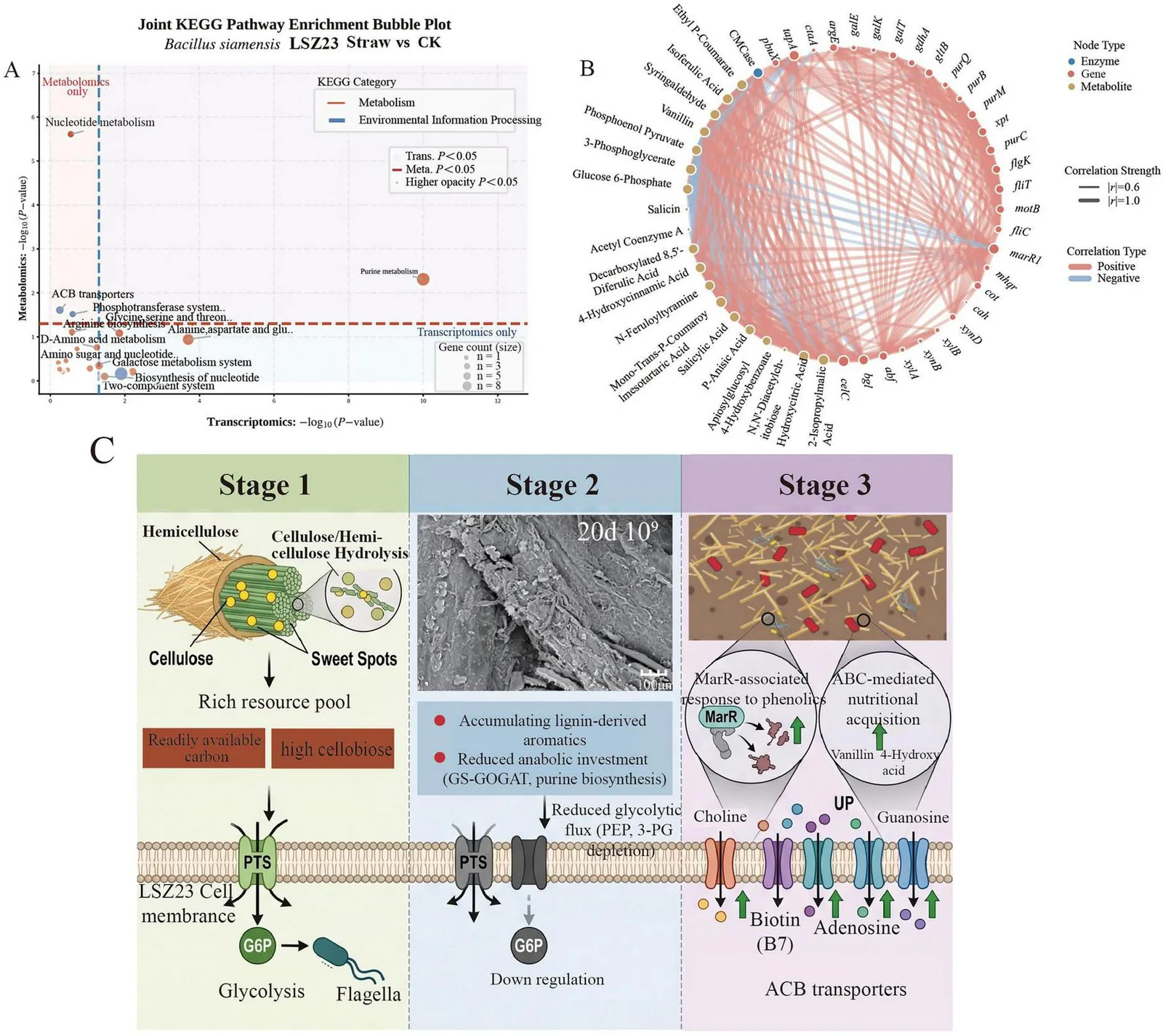
Proposed integrated multiomics model of corn straw degradation by *Bacillus siamensis* LSZ23. **(A)** Joint Kyoto Encyclopedia of Genes and Genomes (KEGG) enrichment of the straw versus control (CK, LB), with the x-axis representing transcriptomic significance [-log_10_(P) ] and the y-axis representing metabolomic significance [−log_10_(P) ]. Dashed lines mark *P* = 0.05; bubble colour denotes KEGG category and size the feature count. Purine metabolism was the only pathway jointly significant in both omics layers. **(B)** Integrated circular correlation network analysis of enzymes, genes and metabolites. The network illustrates the significant correlations calculated using Pearson’s correlation coefficient (—*r*| > 0.8, unadjusted *P* < 0.05). Nodes represent distinct biological variables, colour-coded by their functional categories: blue for enzymes, red for genes (italicised) and gold for metabolites. The size of each node is proportional to its weighted degree (the sum of absolute correlation coefficients) within the network. Edges connecting the nodes indicate significant correlations, with red and blue lines denoting positive and negative correlations, respectively. The width and opacity of the edges scale with the strength of the correlation (—*r*— value). **(C)** Proposed three-phase adaptive model of LSZ23 during corn straw degradation. This model is presented as a working hypothesis generated from multiomics correlation data. Growth (Stage 1): under a replete resource pool, cellobiose is internalised via the phosphotransferase system (PTS) and directed through glucose-6-phosphate (G6P) into glycolysis, with cells remaining flagellated and motile. Reallocation (Stage 2): accumulating lignin-derived aromatics coincide with reduced central glycolytic flux and curtailment of high-cost anabolism (the GS-GOGAT pathway and *de novo* purine biosynthesis). Defence (Stage 3): the data are consistent with a dual-mechanism response, in which upregulated ATP-binding cassette (ABC) transporters may mediate high-affinity acquisition of nutrients and cofactors and in which the concomitant upregulation of five MarR-family regulators parallels the accumulation of lignin-derived phenolics. No functional evidence links these regulators to phenolic sensing or detoxification in LSZ23. Green arrows denote upregulation.

To determine how the enzymatic, transcriptional and metabolic responses were coordinated during straw degradation, a correlation network was constructed using CMCase activity, 28 genes and 20 metabolites (Figure 5B and Supplementary Table 13). Among the 353 significant Pearson correlations (—*r*| > 0.8, *P* < 0.05), 292 (82.7%) were positive and 61 (17.3%) were negative and most —*r*— values exceeded 0.90. The network was organised around a dominant positive module: the cellulolytic genes *celC* and *bglA* and the xylanolytic genes *xylA*, *xylB*, *xynB*, *xynD*, and *abfA* were strongly co-expressed, with *xylA*, *xylB*, and *xynB* almost perfectly correlated (*r* ≥ 0.998) and *celC*–*bglA* reaching *r* = 0.97. CMCase activity fell within this module, correlating positively with *celC* (*r* = 0.95) and *bglA* (*r* = 0.90) as well as with the biofilm- and regulation-associated genes *tapA* (*r* = 0.95) and *marR1* (*r* = 0.96). At the metabolite level, CMCase activity was strongly correlated with lignin-derived aromatics, including syringaldehyde (*r* = 0.998), vanillin (*r* = 0.995), and ethyl p-coumarate (*r* = 0.992), alongside the organic acid hydroxycitric acid (*r* = 0.992); these variables therefore shifted concordantly between the straw and control conditions. In contrast, negative correlations converged on two glycolytic intermediates, phosphoenolpyruvate (PEP) and 3-phosphoglycerate, which together formed the principal negative hub. PEP was inversely correlated with CMCase activity (*r* = −0.996) and with the same aromatic metabolites (e.g., syringaldehyde, *r* = −0.993; vanillin, *r* = −0.988) and 3-phosphoglycerate showed an equivalent trend (CMCase activity, *r* = −0.969). This opposing pattern shows that the lignocellulolytic variables and the lower-glycolysis intermediates moved in opposite directions between the two conditions. With only two conditions compared, this concordance cannot be distinguished from parallel responses to a common stimulus.

## 4 Discussion

Lignocellulose bioconversion is governed not by enzymatic capacity in isolation, but by how a microorganism coordinates the deployment of catabolic enzymes, carbon and nitrogen allocation and chemical stress within the nutrient-limited and increasingly toxic microenvironment of decomposing straw. In this study, *B. siamensis* LSZ23 degraded corn straw with a balanced cellulolytic enzyme system and visible disruption of the lignocellulosic matrix. However, its genomic CAZyme profile alone was insufficient to elucidate the mechanism underlying this hydrolytic performance under substrate-imposed stress. By integrating whole-genome sequencing with comparative genomics, transcriptomics and metabolomics, we infer a regulatory logic that links genetic potential to metabolic output.

Viewed from the perspective of the enzymatic mechanism, these findings describe a four-step catalytic cascade. First, the CE1 esterases, CE4 deacetylases and the α-L-arabinofuranosidase AbfA are positioned to remove feruloyl cross-links, acetyl substituents and arabinofuranosyl side chains from the fibre surface, plausibly releasing the ferulate derivatives that accumulated under straw exposure and the exposed polysaccharide backbones are cleaved by the GH5 endoglucanases and by a GH11 endo-1,4-β-xylanase that ranked among the most strongly induced CAZymes in this study. Notably, this depolymerising capacity is provisioned constitutively, at least at the time point sampled, rather than amplified (the GH5, GH8, GH9, and GH48 genes were transcribed but not significantly induced), placing regulatory control downstream of chain cleavage at this stage. Second, the released oligosaccharides would be captured by the induced cellobiose-specific PTS and by ABC transporters absent from the reference genomes, thereby withdrawing cellobiose, the canonical feedback inhibitor of endo- and exo-acting glucanases, from the extracellular milieu (Lynd et al., 2002). Third, the imported disaccharide would be cleaved as cellobiose-6-phosphate by the induced GH1 6-phospho-β-glucosidase, while cellobiose that is not imported remains accessible to the GH3 β-glucosidases, which are typically extracellular. On this reading, the expanded GH1/GH3 complement, of which the GH1 enzymes are distinguished by high glucose tolerance, represents de-inhibition capacity that sustains upstream hydrolysis rather than redundant gene content. Finally, catalysis is sustained under substrate-imposed stress through non-hydrolytic support, including surface attachment associated with the motility-to-biofilm transition, the reallocation of ATP, NADPH and nitrogen towards cell maintenance and a MarR-associated response characterised by the upregulation of five MarR-family regulators alongside the phenolics that accumulated in the first step. The sections that follow accordingly address the genomic provisioning of this cascade, the reprogramming that sustains it under straw exposure and a working model of how its steps are coupled *in vivo*.

### 4.1 Genomic provisioning of the hydrolytic cascade in LSZ23

Strain LSZ23 displays a functionally balanced cellulolytic profile. Its four canonical activities (CMCase 0.321, β-glucosidase 0.275, avicelase 0.240, and FPase 0.209 U/mL) were distributed evenly rather than skewed towards a single component. This contrasts with strains such as *B. stratosphericus* BHUJPV-H5, which is β-glucosidase dominant (1.33 U/mL; Singh et al., 2019); LSZ23 instead retains measurable activity across all four enzyme classes, including a level of avicelase activity that, although modest in absolute terms, is notable for a *Bacillus* given the scarcity of crystalline cellulose active exoglucanases in this genus. The 19.5% corn straw degradation ratio achieved at 10^9^ CFU/g over 20 days, together with the progressive fibre bundle disruption observed by scanning electron microscopy, indicates that this balanced enzyme system confers an appreciable practical degradation capacity under the tested conditions.

The genomic foundation for this performance was the richness of the LSZ23 CAZyme repertoire, which comprises 570 predicted CAZyme-encoding genes. Because CAZyme repertoire sizes depend strongly on the annotation strategy employed, they are not directly comparable between studies, including those reported for *B. velezensis* (Chen et al., 2022; Zhang et al., 2023). We therefore restrict our comparison to the relative composition of the repertoire within this genome. Among the GH families with a direct role in cellulose saccharification, the 19-copy GH1 complement represents the largest expansion and is markedly expanded relative to the small GH1 complements typically annotated in *B. velezensis* genomes. Genome-wide, the most abundant GH family is GH13 (27 genes), a starch-processing capacity that is independently supported by the two GH13 α-amylases and the CBM26 starch-binding module identified among the orthogroups absent from the four reference genomes (Section 3.5). GH1 β-glucosidases are distinguished from their GH3 counterparts by a deep, narrow active-site pocket associated with markedly higher glucose tolerance (Ketudat Cairns and Esen, 2010; Godse et al., 2021).

Beyond canonical cellulases, the 31 orthogroups absent from the four reference genomes define a further layer of genomic distinctiveness. Their enrichment in oligosaccharide ABC transporter components is mechanistically relevant because the high-affinity import of cellodextrins and xylooligosaccharides allows hydrolysis products to be assimilated directly from the substrate surface, sustaining the cellular energy budget required for continued enzyme secretion in the nutrient-sparse microenvironment of decomposing straw (Nataf et al., 2009). Equally notable is a starch-processing module composed of two GH13 α-amylases, a GH65 glycoside hydrolase and a CBM26 starch-binding domain, which was not detected in any of the four reference genomes. This additional starch-processing capacity in a lignocellulose degrader points to a broadened polysaccharide niche and may reflect adaptation to the chemically heterogeneous humic soil from which LSZ23 was isolated (Earl et al., 2007; Chowdhury et al., 2015).

### 4.2 Non-hydrolytic support that sustains catalysis under straw exposure

Transcriptomic and metabolomic responses to corn straw are consistent with a pattern in which LSZ23 does not broadly amplify its hydrolytic machinery, but instead shows a combination of reduced investment in energy-intensive anabolism and accumulation of lignin-derived phenolics. We note that not every observed change necessarily contributes directly to lignocellulose degradation. Some may reflect non-specific stress or metabolic adjustment. The high C:N ratio of field straw would likely amplify the responses described below, but this was not tested in the present study. Motility and chemotaxis were broadly repressed, three flagellin (*hag*) paralogues, *flgK*, *flgL*, *fliS*, *fliT*, *motB*, and six methyl-accepting chemotaxis protein genes were downregulated, while the matrix anchoring gene *tapA* was induced, a transcriptional pattern resembling that of the *Bacillus* motility-to-biofilm transition, which may exchange costly flagellar synthesis for stable colonisation of the substrate surface (Guttenplan and Kearns, 2013). The transcriptional pattern further suggests that carbon uptake was focused rather than broadened. A PTS cellobiose transporter and a GH1 glucosidase were co-induced, while galactose pathway genes (*galT*, *galE*) were repressed, a pattern compatible with flux being directed towards the cellobiose–glucose route; the depletion of the PTS substrate salicin is consistent with, although not diagnostic of, this redirection in the metabolome. The same economising logic appears applicable to nitrogen and nucleotide metabolism, where the costliest anabolic drains were curtailed: the GS-GOGAT subunits *gltB* and *gltD*, glutamate dehydrogenase *gdhA*, and *de novo* purine biosynthesis (*purF* and *purC*) were all repressed. Because GS-GOGAT consumes ATP and NADPH and supplies glutamate (Gunka and Commichau, 2012), which may reflect a general curtailment of biosynthetic costs rather than a specific nitrogen-sparing response. In a nitrogen-replete medium, ATP and NADPH savings are the more directly relevant interpretation; the nitrogen recovered from repressed pathways would not be limiting under these conditions. As both conditions were cultured in the same nitrogen-replete medium, this differential cannot be attributed to nitrogen availability; moreover, the expression of these genes in *Bacillus* is governed by regulators that integrate carbon and nitrogen status rather than nitrogen supply alone, so their repression is not by itself diagnostic of the cellular nitrogen status.

The second response axis concerns the chemical hazards of lignocellulolysis. As degradation proceeded, lignin-derived phenolics accumulated in the metabolome, including vanillin, syringaldehyde, isoferulic acid, ethyl p-coumarate and 5-hydroxyferulate, alongside the induction of an aromatic compound degradation pathway. Five MarR-family transcriptional regulators were also upregulated under these conditions. MarR-family proteins are known to bind phenolic ligands in other bacteria (Perera and Grove, 2010), but whether the LSZ23 paralogues share this specificity, or whether they modulate efflux or catabolic responses to the accumulated aromatics, remains to be determined.

### 4.3 A working model of how the catalytic steps are coupled *in vivo*

The following model is one interpretation compatible with the observed patterns. It is presented as a working hypothesis for discussion, not as a demonstrated mechanism. CMCase activity, a set of cellulolytic and xylanolytic genes (*celC*, *bglA*, *xylA*, *xylB*, *xynB*, *xynD*, and *abfA*), the biofilm-matrix gene *tapA*, the regulator *marR1* and accumulating lignin-derived aromatics all increased in the straw group relative to the control (Figure 5B). It should be noted that, apart from *tapA* and *marR1*, most of these carbohydrate-active genes did not individually pass the DEG significance threshold (Supplementary Table 10). Notably, this module was mirrored by a negative hub formed by the lower-glycolysis intermediates phosphoenolpyruvate (PEP) and 3-phosphoglycerate, both of which were depleted precisely as CMCase activity and aromatic accumulation rose (PEP–CMCase *r* = −0.996). This inverse relationship points to the redirection of central carbon away from glycolysis as a candidate axis linking the genomic potential of LSZ23 to its metabolic output under straw stress. The network was constructed from six samples representing only two experimental conditions, so the high coefficients obtained largely reflect consistent separation between the straw and control groups rather than continuous covariation and they cannot distinguish direct regulatory coupling from parallel responses to a common stimulus.

The depletion of PEP is consistent with the shift of carbon uptake towards a PTS cellobiose route because the phosphotransferase system is known to consume PEP as its phosphoryl donor to import and phosphorylate incoming sugars. Therefore, an elevated PTS flux is one possible explanation for the lower PEP pool. In parallel, the suppression of GS-GOGAT and *de novo* purine biosynthesis lowers the demand for ATP, NADPH and glutamate-derived nitrogen imposed by the most expensive anabolic sinks, whereas cofactor (NAD^+^, nicotinic acid) and methyl-cycle (S-adenosylhomocysteine) metabolites were increased. Together, these patterns are consistent with a cell that may sustain uptake, maintenance and detoxification while throttling the conversion of that carbon into new biomass, an interpretation that remains to be tested by direct measurement of energy and redox pools. One possible interpretation is that the observed PEP depletion and anabolic downregulation reflect a physiological state in which hydrolytic enzyme secretion and central carbon flux are not maximal, but operate at a rate compatible with concurrent sugar import and aromatic accumulation. This model reconciles the apparent discrepancy of LSZ23, namely that a genome unusually rich in CAZymes (570 predicted CAZyme-encoding genes, including the 19-copy GH1 expansion) may underlie only a focused transcriptional induction and a modest 19.5% degradation ratio. We therefore suggest that the limiting factor is regulatory rather than a shortage of catalytic gene content. Under the conditions examined, aromatic toxicity together with a down-shift of anabolic nitrogen and nucleotide metabolism biases the strain towards survival and persistence rather than bulk hydrolysis. In the field, the high C:N ratio of straw would be expected to reinforce this restraint further, although this prediction remains to be tested under nitrogen-limiting conditions (Figure 5C).

### 4.4 Application potential, limitations, and future perspectives

Although our multiomics analysis characterises LSZ23 as a functionally balanced cellulolytic strain with an expanded CAZyme repertoire, its single-strain degradation efficiency (19.5%) remains modest compared with the > 40% rates recently achieved by multi-species consortia (Wang et al., 2025). This is consistent with the broad recognition that efficient straw decomposition under field conditions relies on the complementary activities of functionally distinct microbial guilds rather than on any single isolate (Harindintwali et al., 2020). Therefore, the optimal translational deployment of LSZ23 is not as a stand-alone inoculant but as a rationally designed consortium partner. Our data provide an initial indication of the functional niche this strain might occupy within such a consortium. LSZ23 appears well-suited to the early hydrolytic phase, providing balanced GH-mediated cellulose cleavage alongside a MarR-associated transcriptional response to the phenolics released during hydrolysis, whose detoxification function remains to be established. In the field, however, straw presents a C:N ratio far higher than that of the nitrogen-replete medium used here and its performance as a stand-alone inoculant may be further constrained by nitrogen availability; the repression of anabolic and purine biosynthetic pathways observed here under nitrogen-replete conditions points to an intrinsic economising tendency that such an environment would be expected to compound. Combining cellulolytic and nitrogen-fixing functions within a single decomposer system is a well-established approach to overcoming the nitrogen limitation that constrains the breakdown of high-C:N residues, thereby increasing both the decomposition rate and the final nitrogen content relative to cellulolytic activity alone. Such a partnership would be predicted to alleviate the metabolic constraints inferred here, thereby accelerating substrate hydrolysis and redirecting the liberated phenolics towards successful humification: lignin-derived phenolics and quinones serve as the aromatic nuclei that polymerise with nitrogenous compounds to form humic substances, the principal route by which straw carbon is stabilised as soil organic matter (Yang et al., 2022).

Three limitations should be acknowledged. First, the regulatory relationships proposed here are inferred from concordant transcriptomic and metabolomic signatures. In particular, it remains to be established that the five MarR-family regulators bind the phenolics detected here and contribute to the tolerance and catabolic responses described above, which would require deletion and complementation together with direct ligand-binding assays. Second, the omics data derive from a single sampling point under a two-condition design, so the temporal ordering implied by the three-phase scheme in Figure 5C remains to be tested by time-resolved sampling. Accordingly, an early and transient induction of the canonical backbone glucanases (GH5, GH8, GH9, and GH48) before the 120-h sampling point cannot be excluded. Densely sampled, time-resolved transcriptomics and metabolomics across the degradation period will be required to resolve the deployment sequence of these enzymes. Third, metabolomic profiling was confined to the intracellular compartment. Although this approach captured the intracellular trade-offs governing enzyme secretion, the exometabolome was not characterised. Future work should therefore incorporate exometabolomic profiling to distinguish secreted metabolites from substrate-derived leachates, which will be critical for predicting microbial interactions when LSZ23 is deployed in synthetic consortia.

Future efforts should prioritise functional testing of whether the five MarR-family regulators sense the phenolics detected here, time-resolved multiomics across the degradation period, characterisation of the response of LSZ23 under defined nitrogen limitation, field-scale validation and the assembly of synthetic microbial consortia. By describing the transcriptional and metabolic changes that accompany degradation in this strain, this study provides a basis for the rational design of straw-degrading microbial consortia.

## 5 Conclusion

We assembled a closed, complete genome of *B. siamensis* LSZ23 and found it to be markedly enriched in carbohydrate-active enzymes, comprising 570 predicted CAZyme-encoding genes typified by a 19-copy expansion of GH1-family β-glucosidases, together with a gene set, absent from the four reference genomes examined, encoding oligosaccharide ABC transporters and starch-processing enzymes. Exposure to corn straw did not broadly induce the secreted backbone-degrading cellulases; rather, it elicited a focused catabolic response centred on the uptake and intracellular processing of hydrolysis products, marked by significant upregulation of a cellobiose-specific PTS transporter, a GH1 6-phospho-β-glucosidase and a GH11 endo-1,4-β-xylanase, whereas the canonical endo- and exo-glucanase genes (GH5, GH8, GH9, and GH48) were not induced and largely trended downward. This uptake-focused induction was accompanied by a physiological switch from flagellar motility to biofilm-associated sessility and by the transcriptional focus of carbon uptake on the cellobiose route. Under these straw-supplemented conditions, the strain also downregulated the energetically costly GS-GOGAT nitrogen-assimilation pathway and *de novo* purine biosynthesis, a signature consistent with the reduction in high-cost anabolism. As the transcriptome was profiled in a nitrogen-replete medium, this response is attributable to straw exposure rather than to nitrogen starvation *per se*. Simultaneously, five MarR-family transcriptional regulators were upregulated in concert with the accumulation of lignin-derived phenolics. Whether this parallel transcriptional response reflects a specific detoxification programme or a general stress reaction remains to be tested. Collectively, these changes accompanied lignocellulose bioconversion under the conditions tested. As these conclusions rest on correlative multiomics evidence obtained at a single time point, the proposed regulatory relationships require functional validation.

## Data Availability

The datasets presented in this study can be found in online repositories. The names of the repository/repositories and accession number(s) can be found in the article/Supplementary material.

## Footnotes

1
https://cloud.majorbio.com
