# Supplementary material for: Multiomics profiling of transcriptional and metabolic responses of Bacillus siamensis LSZ23 to corn straw
Source: Front Microbiol. 2026 Sep 22;17:1935834. doi: 10.3389/fmicb.2026.1935834 (PMC13638650; doi:10.3389/fmicb.2026.1935834)
Supplement: Supplementary file 1 [file Table_1.docx]

Supplementary Material

**Integrated Multiomics Analysis of the Lignocellulose Degradation Mechanism of *Bacillus siamensis* LSZ23**

This supplementary material contains six figures.

Contents

[Fig. S1. Phenotypic characterization of strain LSZ23 using the Biolog GenIII microplate.	2](#_Toc20434)

[Fig. S2. Phylogenetic tree of strain LSZ23.	3](#_Toc28605)

[Fig. S3. Analysis of genome collinearity between strain LSZ23 and reference strains.	4](#_Toc6443)

[Fig. S4. Time course of extracellular CMCase activity of strain LSZ23 in LB supplemented with 2% (w/v) sterilised corn straw.	5](#_Toc4030)

[Fig. S5. PCA score plot.	6](#_Toc26655)

[Fig. S6. PLS-DA score plot.	7](#_Toc11429)

Fig. S1. Phenotypic characterization of strain LSZ23 using the Biolog GenIII microplate. The metabolic profile and chemical sensitivity of strain LSZ23 were assessed across 94 phenotypic tests. The microplate consists of 71 carbon source utilization tests (wells A1 to H4) and 23 chemical sensitivity tests (wells H5 to H12 and specific columns). Color Development: The purple color indicates a positive reaction, resulting from the reduction of a tetrazolium redox dye as the bacteria metabolize the substrates.Interpretation: Solid purple wells: Positive utilization or resistance.Colorless wells: Negative utilization or sensitivity to the chemical. Intermediate/Crossed wells: Borderline or variable reactions requiring further kinetic analysis.

Fig. S2. Phylogenetic tree of strain LSZ23. Relationship of strain LSZ23 with related *Bacillus* species based on 16S rDNA sequences.

Fig. S3. Analysis of genome collinearity between strain LSZ23 and reference strains. This figure presents a comparative analysis of LSZ23 with *B. subtilis* 168, *A. thermocellus* DSM 1313, *B. velezensis FZB42*, and *B. velezensis* SSF6. The red lines represent syntenic blocks, indicating a significant degree of sequence conservation alongside observable local gene rearrangements.

Fig. S4. **Time course of extracellular CMCase activity of strain LSZ23 in LB supplemented with 2% (w/v) sterilised corn straw.** Activity was assayed at 24 h intervals over 6 d. Data are presented as mean ± standard deviation of three independent cultures. The arrow marks 120 h, the point of maximum activity, at which cultures were harvested for transcriptomic and metabolomic profiling.

Fig. S5. PCA score plot. Following dimensionality reduction, samples are plotted as coordinate points along the principal components PC1 and PC2. The distances between these points represent the degree of clustering or dispersion among the samples; shorter distances indicate higher similarity, whereas greater distances indicate larger differences.

Fig. S6. PLS-DA score plot. The PLS-DA score plot is commonly used to visually display the classification performance of the model. A greater degree of separation between the two groups of samples in the plot indicates a more significant classification effect. Component 1: Variance explained by the first principal component; Component 2: Variance explained by the second principal component.
